# Roles of Cu in the Enhanced Thermoelectric Properties in Bi_0.5_Sb_1.5_Te_3_

**DOI:** 10.3390/ma10030251

**Published:** 2017-03-01

**Authors:** Feng Hao, Pengfei Qiu, Qingfeng Song, Hongyi Chen, Ping Lu, Dudi Ren, Xun Shi, Lidong Chen

**Affiliations:** 1 State Key Laboratory of High Performance Ceramics and Superfine Microstructure, Shanghai Institute of Ceramics, Chinese Academy of Sciences, Shanghai 200050, China; haofeng@student.sic.ac.cn (F.H.); songqingfeng@student.sic.ac.cn (Q.S.); chenhy@shanghaitech.edu.cn (H.C.); pinglu@mail.sic.ac.cn (P.L.); rendudi@mail.sic.ac.cn (D.R.); xshi@mail.sic.ac.cn (X.S.); 2 University of Chinese Academy of Sciences, 19 Yuquan Road, Beijing 100049, China

**Keywords:** bismuth telluride, thermoelectric, defect, microstructures

## Abstract

Recently, Cu-containing p-type Bi_0.5_Sb_1.5_Te_3_ materials have shown high thermoelectric performances and promising prospects for practical application in low-grade waste heat recovery. However, the position of Cu in Bi_0.5_Sb_1.5_Te_3_ is controversial, and the roles of Cu in the enhancement of thermoelectric performance are still not clear. In this study, via defects analysis and stability test, the possibility of Cu intercalation in p-type Bi_0.5_Sb_1.5_Te_3_ materials has been excluded, and the position of Cu is identified as doping at the Sb sites. Additionally, the effects of Cu dopants on the electrical and thermal transport properties have been systematically investigated. Besides introducing additional holes, Cu dopants can also significantly enhance the carrier mobility by decreasing the Debye screen length and weakening the interaction between carriers and phonons. Meanwhile, the Cu dopants interrupt the periodicity of lattice vibration and bring stronger anharmonicity, leading to extremely low lattice thermal conductivity. Combining the suppression on the intrinsic excitation, a high thermoelectric performance—with a maximum thermoelectric figure of merit of around 1.4 at 430 K—has been achieved in Cu_0.005_Bi_0.5_Sb_1.495_Te_3_, which is 70% higher than the Bi_0.5_Sb_1.5_Te_3_ matrix.

## 1. Introduction

Utilizing energy in high-efficiency and ecofriendly ways is an urgent task in modern society. However, due to the low efficiency of traditional energy conversion technologies, more than 60% of the total energy is dissipated as waste heat, with the temperatures ranging from ambient temperature to over 1000 °C [1]. Thermoelectric (TE) materials can realize a direct conversion between heat and electricity with the characters of high reliability and zero pollution, and thus provide an alternative choice to use the energy more efficiently [2]. The energy conversion efficiency of a TE material is governed by the figure of merit *zT* = *S*^2^*σT*/*κ*, where *S* is the Seebeck coefficient, *σ* is the electrical conductivity, *T* is the absolute temperature, and *κ* is the thermal conductivity [3]. In order to maximize energy conversion efficiency, a large *S*, a high *σ*, and a low *κ* are required to obtain a high *zT*.

Currently, the best commercial p-type TE materials near room temperature are Bi_2_Te_3_-Sb_2_Te_3_-based alloys developed in the 1950s [4]. Lots of lattice defects are observed in Bi_2_Te_3_-Sb_2_Te_3_-based alloys due to the presence of Bi/Sb atomic disorder distributions and Sb/Te antisites. These point defects can strongly scatter high-frequency heat-carrying phonons to lower lattice thermal conductivities and consequently lead to excellent TE performances. However, the peak *zT*s of these alloys usually appear around room temperature because of their small band gaps (less than 0.2 eV) [5]. At high temperature, the *zT*s are severely degraded due to the enhanced *κ* and quickly decreased *S* caused by the thermal-activated bipolar effect [6,7]. Thus, the applications of these materials for harvesting waste heat as TE power generators are greatly limited. Some effective strategies have been adopted to suppress this thermal-activated bipolar effect. Among them, introducing a tiny amount of Cu into p-type Bi_2_Te_3_-Sb_2_Te_3_-based alloys can effectively decrease the minor carrier (electron) density and significantly weaken the negative effects of minor carriers on the TE performance [8]. High *zT*s, about 1.4 at 400–500 K, have been successfully obtained in Cu_0.005_Bi_0.5_Sb_1.495_Te_3_. Furthermore, the TE modules based on the developed Cu_0.005_Bi_0.5_Sb_1.495_Te_3_ material show a high energy conversion efficiency, about 6% under a temperature gradient of 217 K, superior to the modules using undoped materials.

In spite of the much enhanced TE performance, the roles of Cu in the electrical and thermal transports in p-type Bi_2_Te_3_-Sb_2_Te_3_-based alloys are still not completely clarified. Especially, the occupancy site of Cu atoms is a matter of ongoing debate. Currently, there are two different standpoints. One is that the Cu atoms are in the van der Waals gaps between the two adjacent Te layers in the crystal structure as intercalated ions to introduce excess electrons to the system. As early as 1970, McCarthy et al. [9] found that the external electric field could drive Cu ions into the van der Waals gap. In 2011, Liu et al. [10] proposed the presence of Cu ions in the van der Waals gap of Bi_2_Te_2.7_Se_0.3_ via the characterization and analysis of electrical transport properties and lattice parameters. In 2014, theoretical calculations showed that the formation energy of Cu intercalation in p-type Bi_2_Te_3_ is favorable [11]. Another point is that the Cu atoms prefer to enter the Sb sites due to the small electronegativity and radius difference between Cu and Sb [12,13]. Considering that the valence state of Cu ions is +2, its substitution in the Sb^3+^ site is expected to introduce additional holes to the system, which is completely contrary to the first standpoint. Nevertheless, the substitution of Cu in the Sb sites is well supported by the enhanced *σ* and lowered *S* values obtained in experiments.

In this work, we carried out a further study on the occupancy sites of Cu atoms in p-type Bi_0.5_Sb_1.5_Te_3_ materials. Investigations on the phase compositions, microstructures, electrical and thermal transports, and material stabilities show that the Cu atoms are doped at the Sb sites rather than entering the van der Waals gap in Bi_0.5_Sb_1.5_Te_3_. Then, the roles of Cu atoms on the TE properties were systematically analyzed.

## 2. Results and Discussion

**Figure 1:**
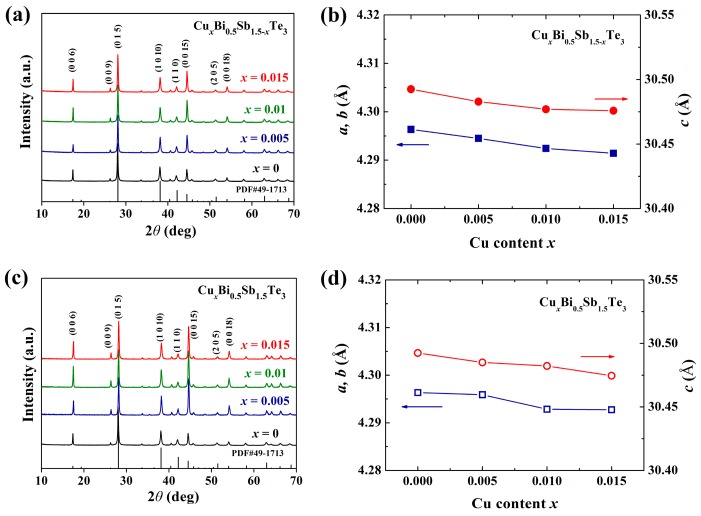
(**a**) X-ray diffraction patterns and (**b**) refined lattice parameters for nominal Cu-doped Cu*_x_*Bi_0.5_Sb_1.5-*x*_Te_3_ (*x* = 0, 0.005, 0.01, 0.015) samples; (**c**) X-ray diffraction patterns and (**d**) refined lattice parameters for nominal Cu-intercalating Cu*_x_*Bi_0.5_Sb_1.5_Te_3_ (*x* = 0, 0.005, 0.01, 0.015) samples.

Figure 1a,c shows the powder XRD patterns for nominal Cu-doped Cu*_x_*Bi_0.5_Sb_1.5-*x*_Te_3_ and nominal Cu-intercalating Cu*_x_*Bi_0.5_Sb_1.5_Te_3_ samples. All the peaks are indexed with the standard Bi_0.5_Sb_1.5_Te_3_ phase (PDF#49-1713). No obvious impurity phase is detected. We further refined the lattice parameters of these Bi_2_Te_3_-Sb_2_Te_3_-based alloys by using the Rietveld method. The results are shown in Figure 1b,d, respectively. If the Cu atoms go into the van der Waals gaps as intercalated ions, the lattice parameter *a* should be scarcely changed, but lattice parameter *c* should be greatly increased [10]. In contrast, if the Cu atoms occupy the Sb sites, both *a* and *c* should be decreased because the atomic radius of Cu (135 pm) is smaller than that of Sb atoms (145 pm). As shown in Figure 1b, the monotonous reduction in *a* and *c* with increasing Cu content strongly suggests that Cu atoms enter the Sb sites in the Cu*_x_*Bi_0.5_Sb_1.5-*x*_Te_3_ samples. Similar lattice contractions are also observed in nominal Cu-intercalating Cu*_x_*Bi_0.5_Sb_1.5_Te_3_ samples (see Figure 1d). Thus, the Cu atoms must be more energy favorable at Sb sites than at the van der Waals gaps in these p-type Bi_2_Te_3_-Sb_2_Te_3_-based alloys.

**Figure 2:**
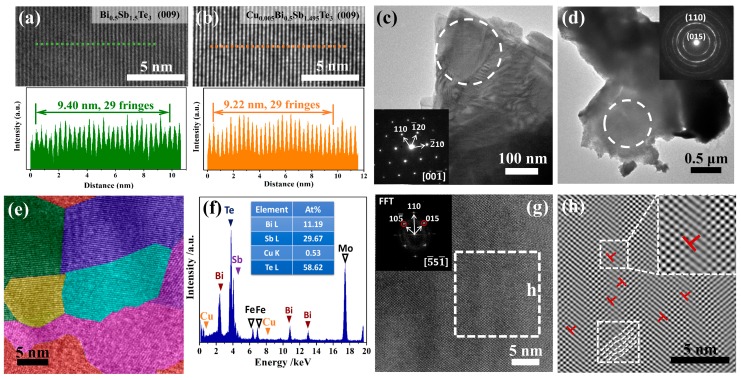
The total distances of 29 fringes in the regular (009) plane TEM images for (**a**) Bi_0.5_Sb_1.5_Te_3_ and (**b**) Cu_0.005_Bi_0.5_Sb_1.495_Te_3_. Low-magnification TEM images for (**c**) Bi_0.5_Sb_1.5_Te_3_ matrix and (**d**) Cu_0.005_Bi_0.5_Sb_1.495_Te_3_. The insets are the corresponding electron diffraction patterns obtained from the circumvented areas. (**e**) Nanograins with coherent grain boundaries in Cu_0.005_Bi_0.5_Sb_1.495_Te_3_; (**f**) The energy dispersive spectrum (EDS) analysis on the circumvented area in Figure 2d. The signals of Mo and Fe are from Mo grids and sample stage; (**g**) HRTEM image of Cu_0.005_Bi_0.5_Sb_1.495_Te_3_. The inset shows the fast Fourier transferred (FFT) image; (**h**) Inverse fast Fourier transferred (IFFT) image of planes (015) and (10$\bar{5}$) in the marked square region in Figure 2g, in which lots of lattice distortions and edge dislocations are observed.

The occupying position of Cu atoms in Bi_0.5_Sb_1.5_Te_3_ samples can also be identified by transmission electronic microscope (TEM) characterization. Figure 2a,b displays the regular (009) plane images for Bi_0.5_Sb_1.5_Te_3_ and Cu_0.005_Bi_0.5_Sb_1.495_Te_3_, respectively. If Cu atoms go to the van der Waals gaps, the (*00l*) plane distance will be expanded, which is the scenario observed in Cu-intercalated n-type Bi_2_Te_2.7_Se_0.3_ by Liu et al. [10]. If Cu atoms go to the Sb sites, the (*00l*) plane distance will shrink. Thus, we compared the distance of 29 fringes in the (009) plane images for both Bi_0.5_Sb_1.5_Te_3_ and Cu_0.005_Bi_0.5_Sb_1.495_Te_3_. The total distance of these 29 fringes in Cu_0.005_Bi_0.5_Sb_1.495_Te_3_ is only 9.22 nm, which is quite smaller than that in Bi_0.5_Sb_1.5_Te_3_ (9.40 nm). This proves that Cu atoms enter the Sb sites rather than the van der Waals gaps in the Cu*_x_*Bi_0.5_Sb_1.5-*x*_Te_3_ samples, which is consistent with the contracted lattice parameters obtained by the above XRD refinement. Interestingly, these Cu atoms at Sb sites induce significant alternations on the microstructures. Figure 2c shows the low-magnification TEM image of the undoped Bi_0.5_Sb_1.5_Te_3_ matrix. The electron diffraction pattern obtained from the circumvented area demonstrates a typical single crystal-like character. This phenomenon is quite different from that in Cu_0.005_Bi_0.5_Sb_1.495_Te_3_. As shown in Figure 2d, the electron diffraction pattern obtained from the circumvented area in Cu_0.005_Bi_0.5_Sb_1.495_Te_3_ shows typical polycrystalline rings, which could be indexed with standard planes in Bi_0.5_Sb_1.5_Te_3_ system. These polycrystalline rings indicate that there are numerous nanograins inside this area. The high-magnification TEM image shown in Figure 2e further proves that the Cu-doped material is indeed composed of nanoscale grains with coherent grain boundaries. The creation of numerous nanograins should be caused by the doped Cu atoms at the Sb sites (see the signal of Cu elements detected by energy dispersive spectrometer in Figure 2f). The discrepancy in atomic radius between Cu^2+^ (135 pm) and Sb^3+^ (145 pm), as well as their different electronegativities and valence states, induce the formation of numerous lattice distortions (see Figure 2g,h). Once the lattice distortions exceed the permitted level, dislocations and grain boundaries will be created to release the strain energy, leading to the formation of numerous nanograins, as shown in Figure 2e. These lattice defects will strengthen the scattering to low-frequency heat-carrying phonons, which is expected to greatly lower the lattice thermal conductivity. Theoretically, various kinds of defects—such as antisite defect, dislocation, and/or vacancies—may exist in Bi_2_Te_3_-Sb_2_Te_3_-based materials [14]. However, in this study, we used a long annealing process time to try to obtain samples near the equilibrium state. In such a case, the formation of various defects is greatly suppressed. All our data show that the Cu atoms are mainly doped at the Sb sites and promote nanocrystallization.

**Figure 3:**
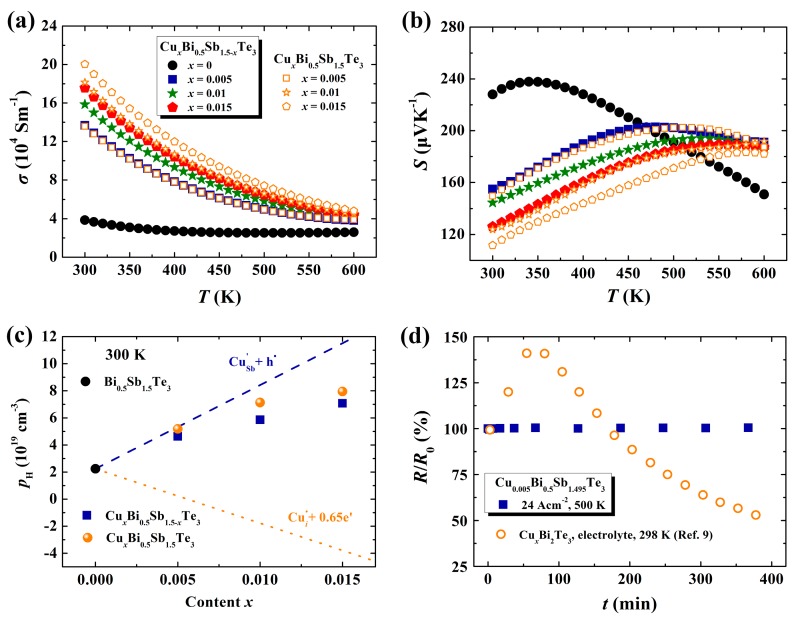
Temperature dependences of (**a**) electrical conductivity *σ* and (**b**) Seebeck coefficient *S* for nominal Cu-doped Cu*_x_*Bi_0.5_Sb_1.5-*x*_Te_3_ samples and nominal Cu-intercalated Cu*_x_*Bi_0.5_Sb_1.5_Te_3_ samples. The *σ* and *S* data for Cu*_x_*Bi_0.5_Sb_1.5-*x*_Te_3_ (*x* = 0, *x* = 0.005) are taken from [8]; (**c**) The measured Hall hole concentration *p*_H_ at 300 K as a function of the nominal Cu content *x* for all Cu*_x_*Bi_0.5_Sb_1.5-*x*_Te_3_ and Cu*_x_*Bi_0.5_Sb_1.5_Te_3_ samples. The dashed and dotted lines represent the predicated $p_{H}$ curves by assuming the Cu atoms at Sb sites and at the van der Waals gap, respectively; (**d**) Relative resistance *R*/*R*_0_ for Cu_0.005_Bi_0.5_Sb_1.495_Te_3_ sample under the current density of 24 Acm^−2^ at 500 K. The data of Cu-intercalated p-type Bi_2_Te_3_ reported by McCarthy et al. are also shown for comparison [9].

The bismuth telluride-based materials possess a typical layered crystal structure, and thus the sintered polycrystalline samples usually show oriented plane alignment and anisotropic TE transport properties [15]. In this manuscript, we will just show the electrical and thermal transports along the direction parallel to the sintering press because this direction shows better TE properties. Figure 3a–c shows the electrical transport properties for all Cu*_x_*Bi_0.5_Sb_1.5-*x*_Te_3_ samples. With increasing Cu content, the *σ* values are gradually increased. At 300 K, the *σ* for Cu_0.005_Bi_0.5_Sb_1.495_Te_3_ is about 13.7 × 10^4^ Sm^−1^, almost 3.5 times larger than that in the matrix compound. Correspondingly, the *S* values near room temperature are greatly reduced in Cu-containing samples. Moreover, in the Cu*_x_*Bi_0.5_Sb_1.5-*x*_Te_3_ system, the peak temperature of *S* is shifted to higher temperatures for the sample with greater Cu content. In p-type Bi_0.5_Sb_1.5_Te_3_ materials, if Cu atoms substitute the Sb atoms, the hole concentration should be enhanced because Cu has fewer electrons in the outermost orbital than Sb (see the dashed line in Figure 3c). This speculation is consistent with the measurement results (square symbols) presented in Figure 3c, which shows that the Hall hole concentration $p_{H}$ increases when increasing the Cu-doping content. For comparison, we also show the *S*, *σ*, and $p_{H}$ data for the nominal Cu-intercalated Cu*_x_*Bi_0.5_Sb_1.5_Te_3_ samples in Figure 3a–c. Furthermore, the measured electrical transports in Cu*_x_*Bi_0.5_Sb_1.5_Te_3_ go against the expectation that the Cu atoms enter the van der Waals gap and introduce additional electrons (0.65 electrons per Cu atom) to reduce $p_{H}$ and *σ* (see the dotted line in Figure 3c) [9]. Thus, the presented electrical transport properties further prove that Cu atoms are doped at the Sb sites in p-type Bi_0.5_Sb_1.5_Te_3_. These Cu-dopants can suppress the intrinsic excitation and shift its occurring temperature to high temperatures, thereby leading to the improved *S* values in Cu-containing samples above 500 K (see Figure 3b).

In bismuth telluride-based materials, the intercalated Cu atoms in the van der Waals gap usually form very weak interactions with the nearby Te atoms because the Te atoms do not have extra free electrons. McCarthy et al. [9] found that the external electrical field could easily drive the Cu ions into the van der Waals gap of p-type Bi_2_Te_3_. However, these Cu ions will quickly diffuse out from the material when the external electrical field is removed. Interestingly, in the present Cu*_x_*Bi_0.5_Sb_1.5-*x*_Te_3_ samples, the doped Cu atoms at the Sb sites have quite high stability under external electric field. We have stressed an external current of 24 Acm^−2^ on the Cu_0.005_Bi_0.5_Sb_1.495_Te_3_ sample at 500 K to check its stability. If Cu atoms are intercalated in the van der Waals gap, such a large current density will definitely drive the Cu ions out from the van der Waals gap and then change its resistivity. This method has been successfully applied to characterize the Cu-ion migration behavior in Cu_2_S ionic conductor [16]. As shown in Figure 3d, under a current density of 24 Acm^−2^, the resistance of the Cu_0.005_Bi_0.5_Sb_1.495_Te_3_ sample is scarcely changed, proving that the Cu atoms have high stability under an external electric field. The Cu atoms occupying Sb sites could form strong chemical bonds with surrounding Te atoms, resulting in the observed high stability.

The tiny amount of Cu dopants in Bi_0.5_Sb_1.5_Te_3_ scarcely changes the band structure. In principle, the density-of-states effective mass m* that reflects band structure can be simulated by the single parabolic (SPB) model as shown below [17]:

(1) $$S=\frac{k_{B}}{e}\left(\frac{\left(5/2+\lambda \right)F_{3/2+\lambda }\left(\eta \right)}{\left(3/2+\lambda \right)F_{1/2+\lambda }\left(\eta \right)}-\eta \right)$$

(2) $$F_{j}\left(\eta \right)=\;\int_{0}^{\infty }\frac{\xi^{j}d\xi }{1+\exp\left(\xi -\eta \right)}$$

(3) $$p=4\pi {\left(\frac{2m^{*}k_{B}T}{h^{2}}\right)}^{3/2}F_{1/2}\left(\eta \right)$$

**Figure 4:**
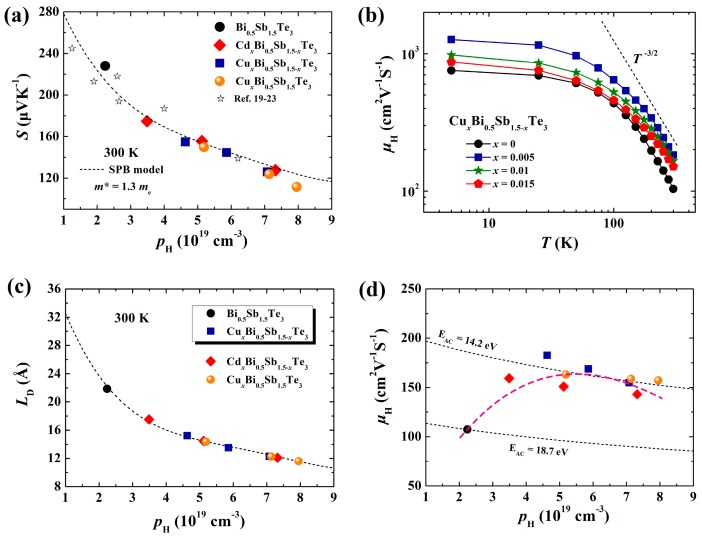
(**a**) The theoretical and experimental Pisarenko plots for Cu-doped Bi_0.5_Sb_1.5_Te_3_ samples. The data of Cd-doped Bi_0.5_Sb_1.5_Te_3_ samples and some other Bi_0.5_Sb_1.5_Te_3_ samples previously reported [19,20,21,22,23] are also shown for comparison; (**b**) temperature dependence of Hall mobility $\mu_{H}$ for Cu-doped samples; (**c**) the Hall hole concentration *p*_H_ dependences of the calculated Debye screen length *L*_D_; and (**d**) the mobility $\mu_{H}$ for Cu-doped Bi_0.5_Sb_1.5_Te_3_ samples. The data of Cd-doped Bi_0.5_Sb_1.5_Te_3_ samples are included for comparison. The dashed line in (**d**) is provided as a guide for eyes. The dotted lines in (**d**) represent the theoretical trends predicted by acoustic phonon scattering mechanism with different deformation potential *E*_AC_.

Here, *k*_B_ is the Boltzmann constant, *e* is the elementary charge, *η* is the reduced Fermi level, and *λ* is the scattering parameter and taken as −1/2 for electron-phonon scattering [18]. *F*_j_ is the Fermi integrals calculated by Equation (2). In Equation (3), *p* is the hole concentration and taken as the measured *p*_H_ value, and *h* is the Planck constant. Figure 4a presents the theoretical Pisarenko plot at 300 K by assuming m* = 1.3*m*_e_ (*m*_e_ is the inertia mass of electron). The experimental *S* data for Cu-doped Bi_0.5_Sb_1.5_Te_3_ samples, Cd-doped samples, and some other Bi_0.5_Sb_1.5_Te_3_ samples previously reported will fall on this calculated line [19,20,21,22,23], suggesting that all samples possess similar m*. This is reasonable, considering the very tiny amount of Cu- or Cd-doping content used in the present study. These dopants just shift the Fermi level downward without obviously changing the band structure near the Fermi level.

The existence of numerous nanograins in Cu-doped samples is expected to strengthen the carrier scattering and reduce the carrier mobility ($\mu_{H}$). Surprisingly, Figure 4b shows that the Cu-doped samples possess much higher $\mu_{H}$ than the undoped Bi_0.5_Sb_1.5_Te_3_. At room temperature, $\mu_{H}$ for Cu_0.005_Bi_0.5_Sb_1.495_Te_3_ is 182.6 cm^2^V^−1^S^−1^, which is about 70% higher than that of the undoped Bi_0.5_Sb_1.5_Te_3_. Especially, such enhanced $\mu_{H}$ values are observed over the entire measured temperature range. These enhanced carrier mobilities might benefit from the weakened ionized impurity scattering and acoustic phonon scattering. Generally, the total mobility *μ*, including these two scattering mechanisms, satisfy the Matthiessen’s rule [24]:

(4) $$\frac{1}{\mu }=\frac{1}{\mu_{I}}+\frac{1}{\mu_{\mathrm{AC}}}$$

where $\mu_{I}$ and $\mu_{\mathrm{AC}}$ is the mobility for ionized impurity scattering and acoustic phonon scattering, respectively. Even in the Bi_0.5_Sb_1.5_Te_3_, the ionized impurity scattering still exists, because there are numerous intrinsic antisite defects (${\mathrm{Sb}}_{\mathrm{Te}}^{\prime }$) or vacancies in the material due to their low formation energies [14]. Generally, the corresponding mobility ($\mu_{I}$) of ionized impurity scattering could be expressed as [25]:

(5) $$\mu_{I}=\frac{64\sqrt{\pi }\varepsilon^{2}{\left(2k_{B}T\right)}^{\frac{3}{2}}}{N_{I}e^{3}\sqrt{m_{s}^{*}}f\left(L_{D}\right)}$$

Herein, *ε* is the dielectric constant and taken as 75 [26]. The relation between density-of-states effective mass m^*^ and single valley effective mass $m_{s}^{*}$ satisfies $m^{*}=N_{v}^{2/3}m_{s}^{*}$, where *N_v_* is valence band degeneracy and taken as 6 for Bi_2_Te_3_-based materials [5,27]. *N*_I_ is the ionized impurity concentration. Dimensionless $f\left(L_{D}\right)$ in Equation 5 can be expressed as:

(6) $$f\left(L_{D}\right)=\ln\left(\frac{12m_{s}^{*}k_{B}TL_{D}^{2}}{\hbar^{2}}+1\right)-\frac{12m_{s}^{*}k_{B}TL_{D}^{2}}{12m_{s}^{*}k_{B}TL_{D}^{2}+\hbar^{2}}$$

where ℏ is reduced Planck constant. $L_{D}$ is Debye screen length, with the formula:

(7) $$L_{D}=\sqrt{\frac{\varepsilon k_{B}T}{pe^{2}}}$$

The shorter *L*_D_ means a smaller Coulomb potential around ionized impurity and thereby a weaker effect on the carrier transports [25]. Based on the Equations (5)–(7), $\mu_{I}$ is simultaneously determined by the ionized impurity concentration *N*_I_ and Debye screen length *L*_D_. As shown in Figure 4c, the *L*_D_ value in the Bi_0.5_Sb_1.5_Te_3_ sample is around 21.9 Å at 300 K. However, in the Cu*_x_*Bi_0.5_Sb_1.5-*x*_Te_3_ samples, the *L*_D_ values are reduced to 12.3–15.2 Å, depending on the hole concentrations. Thus, although Cu-doping slightly increases *N*_I_, the reduced *L*_D_ still leads to the weakened ionized impurity scattering to the carriers.

Besides the ionized impurity scattering, the acoustic phonon scattering to the carriers is also weakened in the Cu-doped samples. As shown in Figure 4b, the acoustic phonon scattering ($\mu_{H}$ ∝ *T*^−3/2^) is the dominant scattering mechanism for all Cu*_x_*Bi_0.5_Sb_1.5-*x*_Te_3_ samples around room temperature. The mobility ($\mu_{\mathrm{AC}}$) for acoustic phonon scattering in the SPB model can be written as [27]:

(8) $$\mu_{\mathrm{AC}}=\frac{{\left(8\pi \right)}^{\frac{1}{2}}e\hbar^{4}\rho \nu^{2}}{3E_{\mathrm{AC}}^{2}{\left(m_{s}^{*}\right)}^{5/2}{\left(k_{B}T\right)}^{3/2}}\psi \left(\eta \right)$$

where *ρ* is density, *ν* is the longitudinal velocity of sound and taken as 2884 m/s [28], and *E*_AC_ is the deformation potential, which is generally used to evaluate the intensity of electron–phonon interaction. ψ(η) in Equation (8) is a monotonous decreasing function with reduced Fermi level (*η*) or hole concentration. It can be expressed as:

(9) $$\psi \left(\eta \right)=\frac{3\sqrt{\pi }}{4}\frac{2\lambda +3/2}{\lambda +3/2}\frac{1}{\Gamma \left(\lambda +5/2\right)}\frac{F_{2\lambda +1/2}\left(\eta \right)}{F_{\lambda +1/2}\left(\eta \right)}.$$

Herein, the increased hole concentration by Cu doping reduces ψ(η) and thus carrier mobility with the magnitude within 9%–25% as compared with undoped matrix. However, by using the $\mu_{H}$ data at 300 K, Equations (8) and (9) (see Figure 4d) show that Cu-doping reduces the deformation potential *E*_AC_ from 18.7 eV to 14.2 eV, which could enhance mobility by more than 70%. Thus, these two effects show that the interaction between carriers and phonons is greatly lessened in Cu-doped samples. Combining the weakened ionized impurity scattering mentioned above, the enhanced carrier mobilities, observed in Figure 4b, in Cu-doped samples can be well explained. As shown in Figure 4c–d, the reduced *L*_D_ and *E*_AC_ can also account for the abnormal enhanced carrier mobilities in the Cd-doped Cd*_x_*Bi_0.5_Sb_1.5-*x*_Te_3_ system.

**Figure 5:**
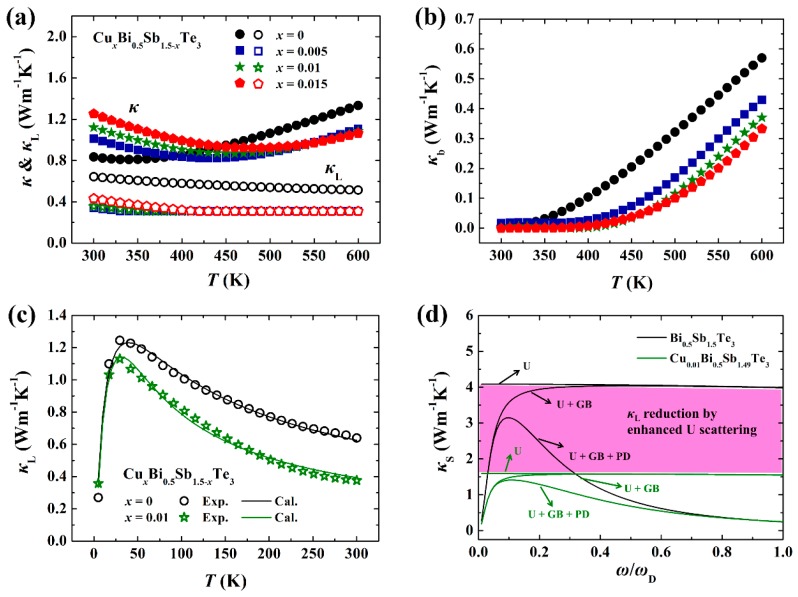
Temperature dependences of (**a**) total thermal conductivity *κ* (and lattice thermal conductivity $\kappa_{L}$) and (**b**) bipolar thermal conductivity $\kappa_{b}$ for Cu*_x_*Bi_0.5_Sb_1.5-*x*_Te_3_ samples. The data for *x* = 0 and *x* = 0.005 are taken from [8]; (**c**) Low-temperature $\kappa_{L}$ for Bi_0.5_Sb_1.5_Te_3_ and Cu_0.01_Bi_0.5_Sb_1.49_Te_3_. The solid lines are the fitting results by Debye model; (**d**) Contribution of phonon–phonon Umklapp scattering (U), point defects scattering (PD), and grain boundary scattering (GB) to the total $\kappa_{L}$ for Bi_0.5_Sb_1.5_Te_3_ and Cu_0.01_Bi_0.5_Sb_1.49_Te_3_ at 300 K. The color-filled area represents the reduction of $\kappa_{L}$ by enhanced U scattering in Cu_0.01_Bi_0.5_Sb_1.49_Te_3_.

Figure 5a displays the temperature dependence of total thermal conductivity *κ* for Cu-doped Cu*_x_*Bi_0.5_Sb_1.5-*x*_Te_3_ samples. The *κ* includes three parts, termed as the carrier thermal conductivity (*κ*_e_), lattice thermal conductivity ($\kappa_{L}$), and bipolar thermal conductivity ($\kappa_{b}$). *κ*_e_ is calculated according to Wiedemann–Franz law (*κ*_e_ = *LσT*, where *L* is the Lorenz number and taken as 1.6 × 10^–8^ V^2^K^–2^) [29]. In this system, the substitution of Cu at the Sb sites significantly improves the electrical conductivity by simultaneously enhancing $p_{H}$ and $\mu_{H}$. Thus, the *κ*_e_ in Cu-doped samples is much higher than that in Bi_0.5_Sb_1.5_Te_3_ matrix. Then, although the $\kappa_{L}$ values in Cu-doped samples are reduced, the total *κ* near room temperature is enhanced. Because Bi_0.5_Sb_1.5_Te_3_ has a very narrow band gap, around 0.2 eV, electrons are easily excited from the valence band to conduction band to form hole–electron pairs. The recombination of these hole–electron pairs releases extra heat and induces the remarkable $\kappa_{b}$ in the Cu-free Bi_0.5_Sb_1.5_Te_3_ sample above 350 K. Doping Cu into the system effectively suppresses the $\kappa_{b}$ via reducing minor carrier concentration (electrons) [8], which can account for the observed low *κ* values at high temperature in Cu-doped Bi_0.5_Sb_1.5_Te_3_ samples.

The very low $\kappa_{L}$ values approaching the theoretical minimum value (0.31 Wm^−1^K^−1^) in Cu-doped Bi_0.5_Sb_1.5_Te_3_ samples are very interesting [30]. We fitted the low-temperature $\kappa_{L}$ of Bi_0.5_Sb_1.5_Te_3_ and Cu_0.01_Bi_0.5_Sb_1.49_Te_3_ by the Debye model, in which $\kappa_{L}$ is expressed by [31]:

(10) $$\kappa_{L}=\;\frac{k_{B}}{2\pi^{2}\nu }(\frac{k_{B}}{\hbar }{)}^{3}T^{3}\int_{0}^{\theta_{D}/T}\frac{x^{4}e^{x}}{\tau_{t}^{-1}{\left(e^{x}-1\right)}^{2}}dx$$

and the spectral lattice thermal conductivity *κ*_s_ can be expressed by [32]:

(11) $$\kappa_{s}=\;\frac{k_{B}}{2\pi^{2}\nu }(\frac{k_{B}}{\hbar }{)}^{3}T^{3}\frac{x^{4}e^{x}}{\tau_{t}^{-1}{\left(e^{x}-1\right)}^{2}}$$

where average sound speed *ν* is taken as 2930 m/s [33]. In this equation, $x=\hbar \omega /k_{B}T$, and *ω* is the phonon frequency. The total relaxation time for Bi_2_Te_3_-based materials is given by:

(12) $$\tau_{t}^{-1}=\;\tau_{B}^{-1}+\tau_{P}^{-1}+\tau_{U}^{-1}=\frac{\nu }{d}+P\omega^{4}+U\omega^{2}Te^{-\theta_{D}/3T}$$

where *θ*_D_ is Debye temperature and taken as 160 K [33]. $\tau_{B}$, $\tau_{P}$, and $\tau_{U}$ are the relaxation time for grain boundary scattering (GB), point defect scattering (PD), and phonon–phonon Umklapp scattering (U), respectively [34]. *d* is the grain size, and *P* and *U* are the coefficients to evaluate the intensity of each phonon scattering mechanism. By fitting the low-temperature $\kappa_{L}$, these parameters can be obtained (see Table 1). Figure 5d shows the integral area of reduced $\kappa_{L}$ by each individual scattering mechanism. Because the exotic Cu atoms interrupt the periodicity of lattice vibration (see the TEM results shown in Figure 2) and bring stronger anharmonicity, the integral area of reduced $\kappa_{L}$ by U process is greatly reduced. Thus, the total $\kappa_{L}$ in Cu_0.01_Bi_0.5_Sb_1.495_Te_3_ is much lower than the Cu-free Bi_0.5_Sb_1.__5_Te_3_.

**Figure 6:**
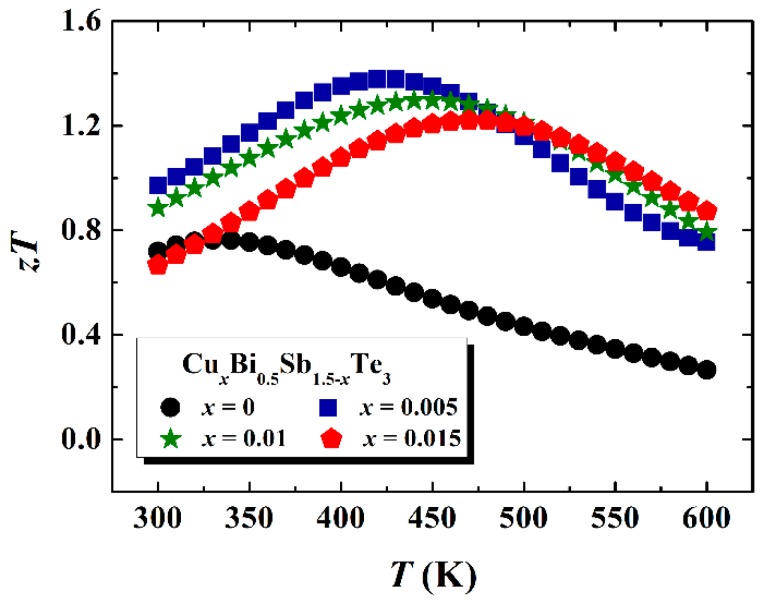
Temperature dependence of figure of merit *zT* for Cu-doped samples. The data for *x* = 0 and *x* = 0.005 are taken from [8].

Figure 6 shows the temperature dependence of TE figure of merit *zT* for Cu-doped Cu*_x_*Bi_0.5_Sb_1.5-*x*_Te_3_ samples. The undoped Bi_0.5_Sb_1.5_Te_3_ matrix displays a maximum *zT* around 0.8 at 330 K. Due to the combined effects of enhanced hole concentration, improved carrier mobility, reduced lattice thermal conductivity, and bipolar thermal conductivity, the maximum *zT* is significantly improved to 1.4. In addition, the suppressed intrinsic excitation in Cu-doped Cu*_x_*Bi_0.5_Sb_1.5-*x*_Te_3_ successfully shifts the peak *zT* towards high temperatures of 430–470 K. Combining the good stability under electric field, these Cu-doped Cu*_x_*Bi_0.5_Sb_1.5-*x*_Te_3_ samples show great potential in applications such as low-grade waste heat recovery.

## 3. Materials and Methods

High purity elements Bi (99.999%), Sb (99.999%), Te (99.999%), Cu (99.999%), and Cd (99.999%) were used to synthesize the samples according to the stoichiometric composition Cu*_x_*Bi_0.5_Sb_1.5-*x*_Te_3_ (*x* = 0.005, 0.01, 0.015,) and Cd*_x_*Bi_0.5_Sb_1.5-*x*_Te_3_ (*x* = 0.005, 0.01, 0.015). The samples with the compositions of Cu*_x_*Bi_0.5_Sb_1.5_Te_3_ (*x* = 0.005, 0.01, 0.015) were also prepared in this study. These elements were firstly sealed into quartz tubes in vacuum, and then melt at 1373 K for 12 h. After quenching into a water bath, they were placed into furnace again and annealed at 673 K for 5 days. Subsequently, the grown ingots were ground to fine powders in agate mortar by hand. These obtained powders were then consolidated by spark plasma sintering (SPS) to form dense cylinders with a height of about 12 mm, which are used to characterize the TE properties along the directions parallel and perpendicular to the pressing. During the sintering procedures, the powders were filled into graphite die and held at 683 K for 10 min under a pressure of 50 MPa. High densities (>98% of the theoretical density) for all sintered samples were obtained.

X-ray diffractometer (XRD, D8 ADVANCE, Bruker Co. Ltd., Karlsruhe, Germany) was used to analyze phase purity for powder samples. The microstructures of the prepared powder samples were also observed by high-resolution transmission electronic microscope (HRTEM, JEOL 2100F, Tokyo, Japan). The chemical composition was analyzed by equipped energy dispersive spectrometer (EDS, Oxford Instruments plc, Oxfordshire, UK). Electrical conductivity (*σ*) and Seebeck coefficient (*S*) above the room temperature were measured by using commercial ZEM-3 (ULVAC Co. Ltd, Kanagawa, Japan) apparatus. The stability under large current density was evaluated by in situ measured resistance in a modified thermal dilatometer (DIL 402c, Netzch Co. Ltd., Selb, Germany). Thermal conductivity (*κ*) is the product of thermal diffusivity (*D*), heat capacity (*C*_p_), and density (*ρ*). The *D* was measured by commercial instrument (LFA 457, Netzch Co. Ltd., Selb, Germany) based on laser flash method. The *C*_p_ was measured by using differential scanning calorimetry (DSC 200 F3, Netzch Co. Ltd., Selb, Germany). The *ρ* was measured by the Archimedes method. The low-temperature Hall coefficient (*R*_H_), *σ*, and *κ* were measured in Physical Property Measurement System (PPMS, Quantum Design, San Diego, CA, USA). *R*_H_ was measured by sweeping magnetic field to 3 T in both positive and negative directions. The Hall carrier concentration is calculated by $p_{H}=1/eR_{H}$, and the mobility ($\mu_{H}$) can be estimated by $\mu_{H}=\;\sigma R_{H}$.

## 4. Conclusions

In this study, the occupancy site of Cu atoms in p-type Bi_0.5_Sb_1.5_Te_3_ materials was studied and their roles on TE properties were illustrated. Via systematical characterizations on the lattice parameters, microstructures, electrical transports, and material stabilities, the possibility of Cu intercalation in p-type Bi_0.5_Sb_1.5_Te_3_ materials has been excluded and the position of Cu is identified as doping at the Sb sites. These Cu dopants not only increase the hole concentration, but also improve the hole mobility by weakening the acoustic phonon scattering and ionized impurity scattering on carriers. Thus, significantly enhanced electrical conductivities are achieved in Cu-doped Bi_0.5_Sb_1.5_Te_3_ materials. Furthermore, the Cu dopants can significantly reduce the lattice thermal conductivity and bipolar thermal conductivity by inducing nanostructural defects and suppressing the intrinsic excitation. Combining the excellent electrical transport properties and low lattice thermal conductivities, the maximum figure of merit *zT* around 1.4 at 430 K has been achieved in Cu-doped samples, almost 70% higher than undoped Bi_0.5_Sb_1.5_Te_3_ matrix.

## Figures and Tables

**Table 1:** Fitting parameters for low-temperature lattice thermal conductivity by using the Debye model.

Cu*_x_*Bi_0.5_Sb_1.5-*x*_Te_3_	*x* = 0	*x* = 0.01
*d* (µm)	2.05	1.52
*P* (10^-41^ s^3^)	8.06	7.09
*U* (10^-18^ sK^-1^)	9.14	23.50
